# The Management of Hypertensive Emergencies—Is There a “Magical” Prescription for All?

**DOI:** 10.3390/jcm11113138

**Published:** 2022-05-31

**Authors:** Ana-Maria Balahura, Ștefan-Ionuț Moroi, Alexandru Scafa-Udrişte, Emma Weiss, Cristina Japie, Daniela Bartoş, Elisabeta Bădilă

**Affiliations:** 1 Internal Medicine Department, Bucharest Clinical Emergency Hospital, Carol Davila University of Medicine and Pharmacy, 050474 Bucharest, Romania; emma.weiss@umfcd.ro (E.W.); cristina.japie@gmail.com (C.J.); daniela.bartos@umfcd.ro (D.B.); 2 Department of Cardiology, Emergency Institute for Cardiovascular Diseases “Prof. Dr. C.C. Iliescu”, 022328 Bucharest, Romania; stefan.moroi@yahoo.com; 3 Cardiology Department, Bucharest Clinical Emergency Hospital, Carol Davila University of Medicine and Pharmacy, 050474 Bucharest, Romania; alexandru.scafa@umfcd.ro; 4 Department of Cardiology, Colentina Hospital, Carol Davila University of Medicine and Pharmacy, 050474 Bucharest, Romania; elisabeta.badila@umfcd.ro

**Keywords:** hypertension, hypertensive emergency, hypertensive urgency, hypertensive crisis, target organ damage, therapeutic approach

## Abstract

Hypertensive emergencies (HE) represent high cardiovascular risk situations defined by a severe increase in blood pressure (BP) associated with acute, hypertension mediated organ damage (A-HMOD) to the heart, brain, retina, kidneys, and large arteries. Blood pressure values alone do not accurately predict the presence of HE; therefore, the search for A-HMOD should be the first step in the management of acute severe hypertension. A rapid therapeutic intervention is mandatory in order to limit and promote regression of end-organ damage, minimize the risk of complications, and improve patient outcomes. Drug therapy for HE, target BP, and the speed of BP decrease are all dictated by the type of A-HMOD, specific drug pharmacokinetics, adverse drug effects, and comorbidities. Therefore, a tailored approach is warranted. However, there is currently a lack of solid evidence for the appropriate treatment strategies for most HE. This article reviews current pharmacological strategies while providing a stepwise, evidence based approach for the management of HE.

## 1. Introduction

Hypertensive emergencies (HE) are defined by a rapid increase in blood pressure (BP) with a systolic value greater than 180 mmHg and/or a diastolic value greater than 120 mmHg, often associated with neurologic, cardiovascular, or renal injury. This organ involvement is currently known as acute hypertension mediated organ damage (A-HMOD) [1]. However, lower thresholds can be associated with hypertensive emergencies in the case of swift elevations from lower baseline BP. Elevated BP alone does not define a HE, no matter how high the value of BP may be, unless it is associated with acute organ injury, for which immediate BP-lowering interventions are mandatory in order to limit the ongoing damage [2]. New or worsening target organ injury can occur in the cerebral, cardiovascular, hematologic, renovascular, and ophtalmologic systems [3,4,5]. The magnitude of BP rise and the absolute BP values are important from a prognostic and therapeutic perspective, early recognition being crucial [6]. 

The most frequent clinical presentations of HE are stroke (38% from all HE), followed by pulmonary oedema (35%) and coronary syndromes (25%) [7], which highlights the fact that uncontrolled hypertension is a powerful contributor to all major cardiovascular outcomes [8]. Immediate recognition and treatment of HE are both, therefore, mandatory. Even though data to demonstrate that the treatment of HE reduces mortality are lacking [4], left untreated, the one-year mortality rate for patients presenting with HE exceeds 79% [9].

The choice of a therapeutic strategy, including the class of antihypertensive drugs as well as the timeline for BP reduction, varies according to the type of A-HMOD, previous comorbidities, specific drug pharmacokinetics, or possible adverse reactions to a certain drug.

Therefore, a tailored approach is warranted. However, there is currently a lack of solid evidence for the appropriate treatment strategies for most HE. This article reviews current pharmacological strategies while providing a stepwise, evidence based approach for the management of HE.

## 2. Definition and Epidemiology—Setting the Stage

A clutter of terms has been used to describe clinical situations associated with increased BP such as HE, hypertensive urgencies, hypertensive crises, or uncontrolled hypertension. The current terminology, however, has retained only the terms HE and uncontrolled hypertension, which can now describe the whole clinical spectrum of acutely elevated BP [10].

Hypertensive emergencies are defined as situations where a severely elevated BP, usually a systolic value higher than 180 mmHg and/or a diastolic value higher than 120 mmHg, is associated with acute, life-threatening organ damage in any of the following key organs: brain, arteries, retina, kidney, and/or heart [11,12,13,14]. The remaining situations with elevated BP but without A-HMOD are named severe uncontrolled hypertension (U-HTN) [10].

The distinction between these two clinical entities is essential because of the major differences in management and treatment [15]. Briefly, patients presenting with HE should receive immediate care, ideally by pharmacological and nonpharmacological interventions for lowering BP levels, especially through the administration of intravenous drugs and specific treatment protocols for the associated clinical conditions including acute coronary syndrome (ACS), acute heart failure with pulmonary oedema, acute aortic syndrome, hypertensive encephalopathy, ischemic or hemorrhagic stroke, pre-eclampsia/eclampsia [16]. On the other side, U-HTN does not usually generate symptoms of damage (A-HMOD), does not require admission in the hospital and generally can be managed by simply reinstituting or intensifying previously prescribed antihypertensive drug therapy [4].

Whatever the cause or clinical presentation may be, two statements are to be remembered. First, there is a lack of evidence from randomized controlled trials to establish the best therapeutic approach or the optimal drug in specific situations. Secondly, BP reductions should be obtained gradually, in a controlled manner, without compromising organ perfusion [14].

Acutely increased BP is a frequent reason patients visit emergency departments (ED). For instance, in a survey of data collected over three years in 1,290,804 adult patients at 114 acute care facilities, systolic BP values higher than 180 mmHg ware present in almost 14% of the cases [17]. The percentage of HE, was, however, much lower as it involved only one in every 200 patients [18]. This rate has remained stable over the last two decades [19,20,21,22,23,24], but it seems to be higher in developing countries, probably due to poor control of the risk factors.

Several clinical studies have been carried out to identify the most common predisposing factors leading to HE. These studies indicate that the most relevant factors responsible for the rapid elevation of BP are low adherence to antihypertensive drug therapy, discontinuation of BP-lowering drugs, abuse of illicit substances and recreational drugs, and poor control of common risk factors (smoke, obesity, hypercholesterolemia, diabetes mellitus) [14,25]. In a prospective study that included patients with hypertension, nonadherence to medication was the most powerful predictor of HE [26].

The importance of an appropriate therapeutic strategy is underlined by the increased in-hospital and out-of-hospital mortality of patients presenting with HE. Recent studies have reported a 12-month mortality rate ranging from 12% to 38.9% [27]. Moreover, higher BP values generate end-organ damage, worsening long-term prognosis. Therefore, an immediate implementation of a therapeutic strategy to decrease BP values is mandatory in order to limit the extension of organ damage and improve prognosis [28].

## 3. Diagnostic Work-Up to Identify Hypertensive Emergencies

The initial assessment should incorporate anamnestic information and physical examination as well as paraclinical evaluation, including imaging tests. A prompt and fast evaluation is critical to limit morbidity and mortality in hypertensive emergencies.

A rigorous medical history should identify details regarding duration and severity of preexisting hypertension and whether HMOD has been previously identified. Antihypertensive medication, history of BP control, and intake of over-the-counter drugs or illicit drugs are important aspects of the medical history.

Blood pressure should be monitored, both in supine and standing positions, as well as in both arms to assess the possibility of aortic dissection if it is found to be significantly different [29]. Physical examination should search for signs of A-HMOD, and so jugular venous distension, crackles, level of consciousness, focal neurological signs, and signs of meningeal irritation need to be evaluated.

**Figure 1:**
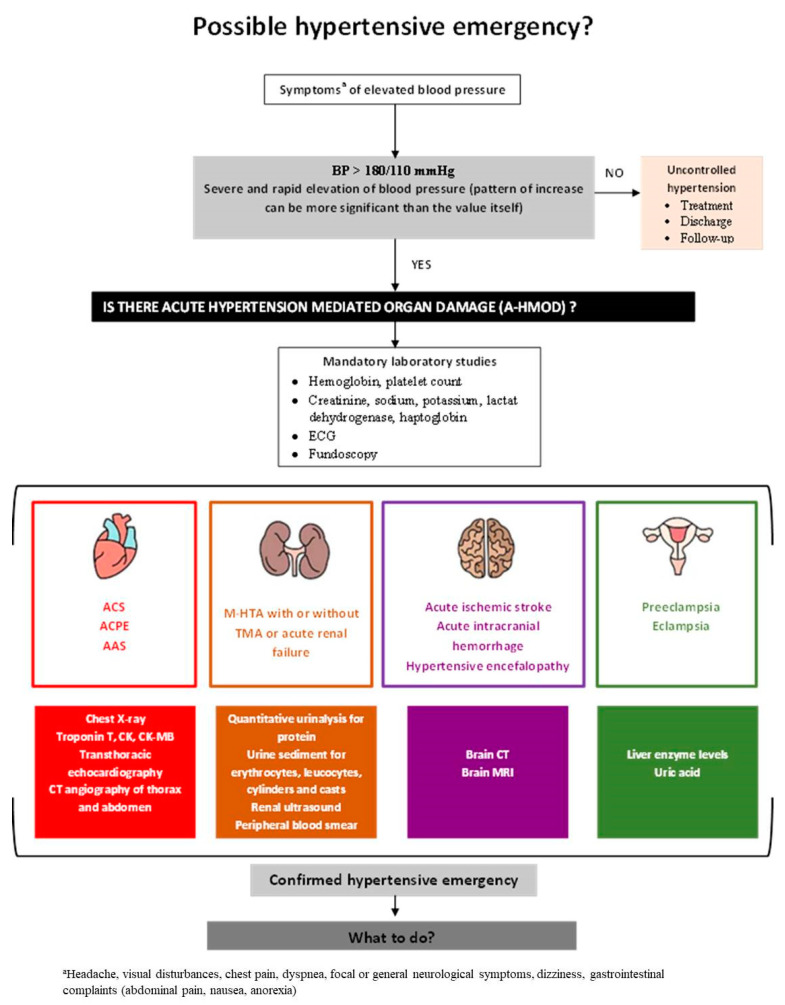
**Diagnostic algorithm of possible hypertensive emergencies**. BP—blood pressure; ECG—electrocardiography; ACS—Acute coronary syndrome; ACPE—Acute cardiogenic pulmonary oedema; AAS—Acute aortic syndrome; M-HTA—Malignant hypertension; CK—Creatine kinase; CK-MB—Creatine kinase-MB; CT—Computer tomography; MRI—Magnetic resonance imaging. According to Table 1 and Figure 2.

All patients with suspected HE will be evaluated through usual investigations such as a basic metabolic panel, complete blood count, electrocardiogram, urinalysis, and chest X-ray. Further specific investigations should be based on symptoms and aligned with each associated condition’s differential diagnosis. For example, a patient presenting with altered mental status and BP > 220/120 mmHg will require brain imaging by computed tomography to assess for intracerebral hemorrhage or hypertensive encefalopathy. If neither is present, and there is no another explanation for the altered mental status, magnetic resonance imaging may be needed [30]. Likewise, patients presenting with chest pain or shortness of breath should be evaluated by troponin and natriuretic peptides. If acute aortic syndrome is suspected, computed tomography angiography of the thorax and abdomen is mandatory (see Figure 1). As indicated in the guidelines, ED-recommended testing is guided by specific clinical presentation. In a study evaluating 423 patients presenting with suspected HE, only 6% of the patients admitted in the ED benefited from a comprehensive and exhaustive evaluation, including fundoscopy, the most complete evaluation being in the case of cardiovascular presentation symptoms [31].

## 4. Management

### 4.1. General Principles

Preventing the occurrence of HE should be as important as the treatment itself. Therefore, implementing the main guideline recommended interventions for hypertension control is the key to such a prevention strategy. The main pillars of HTN control and preventions of the acute rise of BP are substantial lifestyle interventions and pharmacological management. The most important lifestyle changes that should be recommended are salt restriction (<5 g per day); reduction of alcohol consumption (less than 14 units per week for men, and less than 8 units per week for women); weight reduction (body-mass index about 20–25 kg/m^2^ and waist circumference values <94 cm in men and <80 cm in women); a healthy balanced diet with a high intake of vegetables, fruits, fish, and unsaturated fatty acids and a low intake of red meat and saturated fatty acids; constant physical activity (at least 30 min of moderate dynamic exercise on at least 5 to 7 days per week); and cessation of smoking [32]. However, an even more important pillar of the preventive strategy is maintaining medication adherence as it has been shown to be one of the most prevalent risk factors of HE [26]. Moreover, close monitoring of BP and a rapid change of antihypertensive treatment plan when BP control is not attained should be incorporated in the HE preventive strategy [32].

There is currently a lack of evidence from randomized controlled trials regarding the management of HE. The drug of choice, rate, and optimal time for lowering BP are established only from experts’ opinion and retrospective studies [33] and are dictated by the type of organ damage, the presence of comorbidities, or contraindications to a specific drug.

Patients presenting with HE should be admitted to an intensive care unit (ICU) for close monitoring. The target is not to achieve a particular BP value, but to preserve organ perfusion and to prevent hypertensive target organ injury [10]. Comorbidities and target organ involvement influence medical decisions concerning optimal BP, the time to achieve BP control, and the choice for the ideal pharmacological therapy. Intravenous medications are preferred because of their fast onset and ability to titrate, and their generally short half-life [10].

A general goal could be lowering BP in a controlled manner with a systolic BP reduction of no more than 25% within the first hour, followed by a gradual reduction to 160/100 mmHg within the next two to six hours, before cautiously reducing the BP to normal over the next 24 to 48 h [4]. However, exceptions are not rare, such as aortic dissection, pre-eclampsia, or pheochromocytoma, which require rapid BP reduction, while others require less aggressive approaches, such as ischemic stroke.

The most efficient drugs listed for HE treatment include nicardipine, labetalol, esmolol, and clevidipine. Nitroprusside was a pillar of treatment for many decades, but similar efficacy is shared by nicardipine and clevidipine, which are easier to titrate and present without risk of cyanide toxicity [13,34,35,36]. In particular, diuretics are of no use for emergency BP treatment. Their effect on BP is very unpredictable, and most patients do not present hypervolemia. A list of intravenous BP-lowering drugs with their dose, mechanism of action, adverse effects, and contraindications is provided in Table 1.

**Table 1:** Intravenous antihypertensive drugs for the management of hypertensive emergencies. I.v.—intravenous; Kg—kilograms; COPD—Chronic obstructive pulmonary disease; PDE-5 inhibitors—Phosphodiesterase 5 inhibitors.

Intravenous Antihypertensive Drugs for the Management of Hypertensive Emergencies				
Drug	Dose	Mechanism of Action	Adverse Effects	Contraindications
**ESMOLOL**	500 to 1000 μg/kg i.v. bolus in 1 min or 50–250 μg/kg/min continuous i.v. infusion	Cardioselective β1-blocker resulting in decreased cardiac output	Hypotension, Dizziness, Peripheral ischemia, Infusion site reaction, Bradycardia	Sinus bradycardia, Sick sinus syndrome, Second- or third-degree heart block, Heart failure, Cardiogenic shock, Pulmonary hypertension, Asthma, COPD
**LABETALOL**	0.25–0.5 mg/kg i.v. bolus or 2–4 mg/min i.v. infusion, thereafter 5–20 mg/h	Non-selective α1 and β-adrenergic blocker resulting in decreased cardiac output and direct vasodilation	Symptomatic postural hypotension, Flushing, Acute left ventricular failure, Bronchospasm, Bradycardia	Asthma, Heart failure, Second- or third-degree heart block, Cardiogenic Shock, Severe bradycardia
**CLEVIDIPINE**	1–2 mg/h i.v. infusion, increase every 2 min with 2 mg/h i.v. bolus or 15–30 mg/min continuous i.v. infusion	Block L-type calcium channels, which leads to coronary and peripheral vasodilation	Systemic hypotension, Reflex tachycardia	Allergies to soybeans, soy products, eggs or egg products, Defective lipid metabolism, Severe aortic stenosis
**NICARDIPINE**	5 mg/h continuous i.v. infusion, increase dose by 2.5 mg/h every 15 min to a maximum dose of 15 mg/h	Block L-type calcium channels, which leads to coronary and peripheral vasodilation	Dizziness, Flushing, Reflex tahycardia, Nausea, Vomiting, Increased intracranial pressure	Liver failure
**NITROGLYCERINE**	5–200 μg/min continuous i.v. infusion, increase by 5 μg/min every 5 min	Nitric oxide donor	Headache, Reflex tachycardia, Vomiting, Flushing, Methemoglobinemia, Syncope Venodilator	Known history of increased intracranial pressure, Severe anemia, Right-sided myocardial infarction, Concurrent use with PDE-5 inhibitors
**NITROPRUSSIDE**	0.25–10 μg/kg/min continuous i.v. infusion, increase by 0.5 μg/kg/min every 5 min to a maximal dose only for 10 min	Nitric oxide donor Direct arterial and venous dilator	Nausea, Vomiting, Muscle twitching, Thiocyanate intoxication, Methemoglobinemia acidosis, Cyanide poisoning	Concurrent use with PDE-5 inhibitors, Septic shock, Vitamin B12 deficiency
**ENALAPRILAT**	0.625–1.25 mg i.v. bolus every 6 h	Inhibits conversion of angiotensin I to angiotensin II causing vasodilation, reduced aldosterone secretion, inhibiting cardiac and vascular remodeling	Hypotension, Cough, Hyperkaliemia, Cholestatic jaundice	Renal failure in patients with bilateral renal artery stenosis, History of angioedema, Pregnancy and lactation, Acute myocardial infarction
**CLONIDINE**	150–300 μg i.v. bolus in 5–10 min	Agonist of both imidazoline and α2-adrenergic receptors reducing sympathetic outflow from the vasomotor center in the brain and increasing vagal tone	Sedation, Rebound hypertension	
**PHENTOLAMINE**	0.5–1 mg/kg i.v. bolus or 50–300 μg/kg/min continuous i.v. infusion	Non-selective α-adrenergic blocker	Tachyarrhythmias, Orthostatic hypotension, Chest pain	

Pharmacological therapies are available only in about 30% of the diseases identified, and many biological targets for numerous diseases are yet to be identified [37]; therefore, we should use those agents (see Table 1) that have been studied for lowering the BP safely and effectively.

In an intensive care unit setting, nicardipine is a more effective antihypertensive agent than labetalol, with a better adverse effects profile, associated with less hypotension, bradycardia, and atrioventricular block, resulting in lower rates of drug discontinuation [38].

A retrospective study involving 90 patients presenting with acute stroke and hypertension demonstrated that nicardipine is a viable alternative to labetalol, with similar tolerability and a trend towards less variability in BP response following nicardipine [39]. Moreover, in a prospective study evaluating acute BP management in stroke patients showed that nicardipine has a superior therapeutic profile, with a more predictable BP response and less variation than labetalol, but with no differences in clinical outcomes [40].

A study comparing cerebral hemodynamics in malignant hypertension demonstrated that both labetalol and nitroprusside reduce BP adequately, but labetalol reduced both systemic and cerebral vascular resistance, whereas sodium nitroprusside reduced the systemic vascular resistance rather than cerebral vascular resistance with a larger rate of reduction in the middle cerebral artery, which suggests a preferential blood flow to the low resistance systemic vascular bed rather than the cerebral vascular bed [41].

Another study randomized 226 patients with acute hypertension to intravenous nicardipine or intravenous labetalol, which showed that patients treated with intravenous nicardipine are more likely to reach the specified BP goals within 30 min than those treated with intravenous labetalol [42].

Current available data do not provide enough evidence suggesting that one specific intravenous (IV) antihypertensive agent is superior to another, with the possible exception of neurological HE, in which treatment recommendations are more evidence based with dihydropyridine agents such as nicardipine, clevidipine, and α/β blocker—labetalol as the most efficient agents [14].

**Figure 2:**
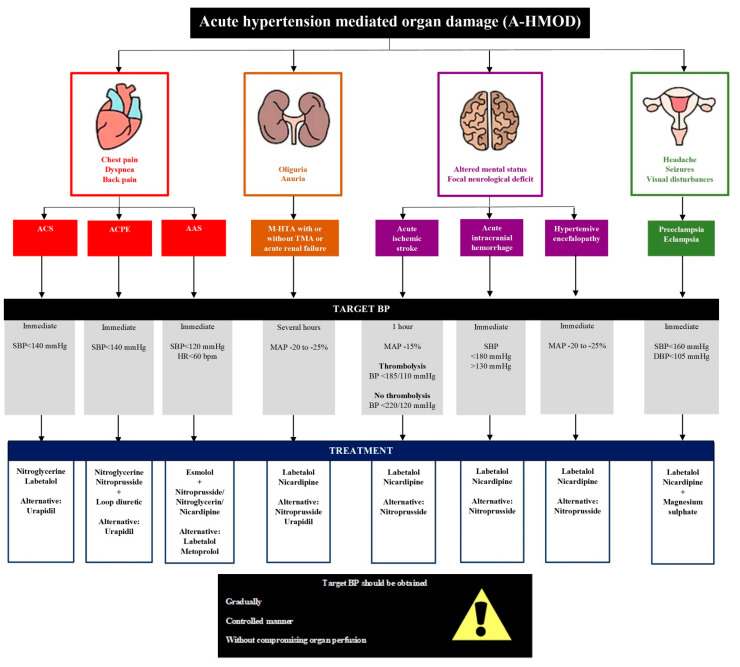
**Treatment of hypertensive emergencies according to the main acute hypertension mediated organ damage (A-HMOD).** ACS—Acute coronary syndrome; ACPE—Acute cardiogenic pulmonary oedema; AAS—Acute aortic syndrome; M-HTA—Malignant hypertension; TMA—Thrombotic microangiopathy; BP—Blood pressure; SBP—Systolic blood pressure; DBP—Dyastolic blood pressure; HR—Heart rhythm; MAP—Mean arterial pressure.

After achieving the target BP, switching to oral therapy is advised. Some clinical situations require different management strategies, as discussed below (See Figure 2).

### 4.2. Hypertensive Encefalopathy

Generally, when a moderate rise in systemic BP occurs, the mechanism of cerebral autoregulation intervenes, resulting in the vasoconstriction of cerebral arterioles in order to preserve a constant blood flow rate to the brain [43]. In the case of rapid elevation of BP, this autoregulatory response is incapable of preventing cerebral hyperperfusion, causing increased intracranial pressure, damage of the blood–brain barrier, and fluid extravasation into the brain tissue, especially in the posterior regions, where BP oscillations are less effectively managed because of lowered sympathetic innervation [44]. On the other hand, when rapid or important BP reduction occurs, patients may experience symptoms of organ hypoperfusion [45]. If no ischemia or hemorrhage is present, clinical and radiological findings gradually disappear once the BP is controlled. A syndrome of posterior reversible encephalopathy can be described in the presence of acute onset headache, seizures, altered consciousness, and visual disturbance occurring in the context of increased BP [43]. 

Thus, a controlled and gradual decrease in BP is the cornerstone of hypertensive encephalopathy treatment. The initial management should be aimed at lowering the mean arterial BP by 20 to 25% in the first hours [10]. A further decrease in mean arterial BP increases the risk of cerebral hypoperfusion. There are currently no clear data as to what the optimal time is to reduce the BP or the most efficient agents to control it. Nicardipine or labetalol are preferred because they can be administered continuously, therefore avoiding major BP fluctuations, which in the set of altered autoregulation can disrupt the normal blood flow [30]. It is, however, contraindicated to administer nitroglycerine in hypertensive encephalopathy because of its venous vasodilatory effect and the potential to increase intracranial pressure, thus worsening cerebral oedema [41]. Nitroprusside can be used as it is a more balanced arterial and venous vasodilator and acts mainly on the systemic rather than the cerebral vascular resistance [41]. 

If not properly treated, hypertensive encephalopathy and posterior reversible encephalopathy syndrome can evolve to cerebral hemorrhage, coma, and death. An appropriate and optimal treatment can lead to full recovery [46], which highlights the central role of an immediate diagnosis and an effective BP-lowering treatment.

### 4.3. Acute Ischemic Stroke

Managing hypertension in acute ischemic stroke can be cumbersome because increased BP can be beneficial as a compensatory physiological regulation to inadequate localized cerebral perfusion pressure while increasing cerebral hemorrhagic risk [47]. Reducing the cerebral perfusion pressure could lead to expansion of the infarction zone as a consequence of further reducing perfusion to ischemic tissues [48]. Thus, higher BP targets are an acceptable goal. This concept is endorsed by several trials investigating the management of hypertension in the acute stroke setting and showing either neutral effects or harm when BP was lowered. However, many trials have excluded patients with severely increased BP (>220 mmHg systolic BP), therefore data are lacking regarding BP-lowering thresholds in such patients. Nonetheless, abstaining from rapidly and significantly decreasing BP in the first 24 h is advisable as some trials have reported negative effects when BP was lowered more than 20% in the acute phase [49,50]. 

Patients with suspected ischemic stroke who are eligible for thrombolysis must benefit from a rapid BP reduction to less than 185/100 mmHg and further maintenance for at least 24 h to reduce the risk of intracranial hemorrhage [48]. However, the systolic BP should not be reduced to a value less than 130–140 mmHg in the first 72 h, while keeping it under 180 mmHg [51]. Intravenous nicardipine, labetalol, and clevidipine have been approved as initial agents in recent stroke guidelines [48]. Nitroprusside can be considered if the target BP is not reached by the former agents or if the diastolic BP is greater than 140 mmHg.

Management of hypertension in patients following successful mechanical thrombectomy is still an area of study, current literature suggesting better outcomes when systolic BP is less than 160 mmHg, or even 140 mmHg [52]. 

For patients not eligible for thrombolysis or thrombectomy, maintaining higher BP is advised, even as high as 220/120 mmHg to maintain cerebral perfusion with potentially reversible ischemia. In the first 24 h, a reduction of less than 15% can be considered safe and reasonable to pursue [51]. Over the next 24 to 48 h, the blood pressure needs to be gradually lowered [53]. Patients presenting with transient ischemic attack should be treated likewise. 

### 4.4. Acute Intracranial Hemorrhage

Blood pressure and coagulopathy management for intracranial hemorrhage and acute ischemic stroke are different; therefore, treatment should be started after the diagnosis is confirmed. Results from different large randomized controlled trials have been unable to formulate specific BP targets for patients suffering from spontaneous intracranial hemorrhage [54,55].

The INTERACT-2 trial, which enrolled patients within 6 h of symptom onset, demonstrated that reducing the systolic BP to less than 140 mmHg, rather than reducing it to only less than 180 mmHg, had slightly better functional outcomes [54]. The ATACH-2 trial compared the same target for systolic BP of less than 140 mmHg vs. less than 180 mmHg, but within 4.5 h of symptom onset. Results showed no difference in mortality or functional outcomes between the two treatment groups, but the aggressive target showed higher rates of renal dysfunction after seven days [55]. However, on a post-hoc analysis of ATACH-2, it was shown that in patients administered antihypertensive therapy within 2 h of symptom onset, the hematoma expansion was lower, with a better functional outcome at three months. Another analysis from INTERACT-2 demonstrated that systolic BP lower than 130 mmHg increased the risk of physical disability compared to systolic BP between 130 mmHg and 140 mmHg [56]. Thus, there is ongoing controversy as to whether lowering systolic BP to less than 140 mmHg is beneficial.

Based on the available data, current guidelines have concluded that in patients presenting with hyperacute intracerebral hematoma (symptoms less than 6 h), lowering BP to less than 140 mmHg (but not lower than 110 mmHg) is advisable as it can lead to a reduction in hematoma expansion. Nonetheless, the magnitude of BP reduction should not be larger than 90 mmHg [51]. 

There is currently a lack of evidence from randomized controlled trials to suggest an ideal drug for hypertension in the context of acute intracranial hemorrhage. Treatment should be started with an intravenous, rapid acting, easily titratable drug, such as clevidipine, labetalol, nicardipine, or urapidil [13]. For instance, in a multicenter comparison of outcomes, nicardipine compared with nitroprusside infusion during the first 24 h after intracranial hemorrhage is associated with reduced risk of in-hospital mortality without any increase in hospitalization cost or length of stay [57].

### 4.5. Acute Coronary Syndrome

Myocardial infarction or unstable angina require an immediate recognition by performing an electrocardiogram and laboratory studies including measuring cardiac enzyme levels, oriented by a detailed medical history. A retrospective data analysis of 236 patients presenting with HE highlighted that patients with elevated cardiac troponin I levels had a nearly three-times higher risk of major cardiovascular or cerebrovascular events at two years follow-up versus patients with normal cardiac troponin I levels [58].

Treatment of acute, ongoing, myocardial ischemia associated with severe BP elevations should be aimed at decreasing LV (left ventricle) preload and afterload while maintaining a low heart rate to provide adequate diastolic filling time and decrease myocardial oxygen demand. These can be achieved with the administration of intravenous nitroglycerin up-titrated for controlling the angina while rapidly lowering the systolic BP to less than 140 mmHg associated with intravenous β-blockers, usually esmolol or labetalol [59], which lower the cardiac output and myocardial oxygen consumption, while reducing the rate of nitroglycerin-induced reflex tachycardia [60]. If β-blockers are contraindicated, diltiazem or verapamil are a reasonable alternative. Nitroprusside should be avoided in acute coronary syndromes because of its unfavorable effect on the distribution of myocardial blood flow during ischemia [61].

The selective α1 blocker urapidil is a good alternative for the management of hypertension in patients with acute coronary syndromes. In patients with ST elevation, myocardial infarction and percutaneous revascularization, urapidil improved coronary flow, myocardial perfusion, and LV function, were beneficial effects that were associated with an increased production of nitric oxide [62].

Patients with acute coronary syndromes commonly receive anti-platelet medication, which increases the risk for cerebral hemorrhage in the presence of elevated BP, thus adequate control of BP values is a mandatory step [45]. Nonetheless, during acute ischemia, a diastolic BP of less than 60 mmHg needs to be avoided as this could lower myocardial perfusion and aggravate the ongoing physiopathologic process. At discharge, a BP < 140/90 mmHg is recommended for all patients, while for patients under 65 years a lower chronic BP should be achieved, in the range of 120–130 mmHg for systolic BP and 70–80 mmHg for diastolic BP [32].

### 4.6. Acute Cardiogenic Pulmonary Oedema

Acute heart failure and acute pulmonary edema are observed in up to 23% of ED visits for acute severe hypertension [20]. These patients require careful management with the immediate goals of reducing the afterload, improving the LV ejection fraction, and resolving the lung congestion. Therefore, the optimal treatment is the association of intravenous loop diuretic (furosemide) associated with nitroprusside or nitroglycerine up-titrated to the highest tolerated dose for decreasing both cardiac preload and afterload [10]. A mineralocorticoid receptor antagonist can be combined with the loop diuretic to prevent hypokalemia [44]. Non-invasive positive-pressure ventilation can also improve hemodynamics by reducing venous return [10]. β-blockers, which reduce cardiac contractility, and hydralazine, which increases cardiac work, are contraindicated in this acute situation [11].

The BP should be lowered by about 20 to 25% within minutes to 1 h, then gradually to 160/100 mmHg within the next 2 to 6 h, and finally returned cautiously to normal over the next 24 to 48 h [11].

A study randomized 104 patients with acute heart failure and hypertension to receive intravenous clevidipine versus standard-of-care intravenous antihypertensive drugs (87% intravenous nitroglycerin or nicardipine). The study showed that the target BP level was reached in 71% of patients treated with clevidipine versus 37% treated with the latter two. Clevidipine was also more effective at improving dyspnea at 45 min [63].

Urapidil is a good alternative to nitroglycerine in patients with acute heart failure. The NITURA study showed a more pronounced decrease in BP and an improved respiratory and metabolic outcome in patients with acute pulmonary edema receiving urapidil versus nitroglycerine [64].

Another prospective study comparing the efficacy of nicardipine and nitroprusside in HE presenting with acute pulmonary edema demonstrated that there were no time-dependent differences between the two groups, suggesting that nicardipine has similar therapeutic efficacy to sodium nitroprusside, at the same time being easier to administer [65].

### 4.7. Acute Aortic Syndrome

The annual incidence of all acute aortic syndromes, which include aortic dissection, is low in the general population, ranging from 4 to 6 cases per 100,000 persons/year, while in people older than 65 years, it increases to about 30 cases per 100,000 persons/year [66]. Moreover, it remains a life-threatening condition with a high mortality rate. The optimal treatment for type A aortic dissection is surgery, whereas type B aortic dissection can be medically treated. About 26% of patients with type A dissection do not survive, even with surgery, but this figure rises to 58% if treated non-surgically because of other comorbidities [66,67].

Medical therapy consists of controlling chest pain and administering the “anti-impulse therapy” represented by the rapid lowering of systolic BP to 100 to 120 mmHg with a concomitant reduction of heart rate to lower than 60 beats per minute [68,69]. This therapy is required to decrease the aortic wall shear stress and to minimize the tendency for the dissection to propagate, as well as decreasing the development of complications (rupture, aneurysmal degeneration). Intravenous β-blockers are the drugs of choice in this situation. Intravenous labetalol, which is a non-selective β-blocker with α- and β-adrenergic blocking effects, can be used for rapid BP reduction [10]. Esmolol, which has a shorter half-life, may be favored in aortic dissection as it allows for rapid correction if hypotension develops [11]. If a significant contraindication to β-blockade is present, calcium channel blockers are a suitable alternative [70]. Nitroprusside or nitroglycerin administered intravenously can be used if the target BP remains elevated but with additional or after β-blockade to prevent reflex tachycardia.

### 4.8. Thrombotic Microangiopathy and Acute Renal Failure

Malignant hypertension (M-HTN) is one the most severe forms of hypertension. As a HE, it can develop in patients with a known history of primary hypertension, but in most cases, up to 60%, it occurs de novo with no differences in signs, symptoms, or long-term survival [71]. The five-year-survival rate has improved dramatically over the last few decades, currently being more than 90% [72].

Thrombotic microangiopathy (TMA) results from hypertensive endothelial injury, which triggers platelets activation, trombi formation, microvessels obliteration, and disseminated intravascular coagulation [10].

The HE involving TMA is similar to thrombotic thrombocytopenic purpura and hemolytic-uremic syndrome. It is important to differentiate this disease from the former two, because antihypertensive treatment will usually improve TMA and associated renal failure, but thrombotic thrombocytopenic purpura and hemolytic-uremic syndrome might require a specific treatment, such as plasma exchange and immune suppression in thrombotic thrombocytopenic purpura [73] or eculizumab in hemolytic-uremic syndrome [74].

The first-line pharmacological treatment is with labetalol and nicardipine; alternatively, nitroprusside and urapidil can be used as a safe and effective treatment [10]. The cornerstone of treatment is the complete blocking of the renin-angiotensin system (RAS). These patients can be treated with oral medication without delay, especially RAS blockers, but at a very low dose because they usually present with hypovolemia, with a forced titration at every 6 h, which is a safe, effective, and tolerated maneuver. Saline infusion may be used to avoid a drastic decrease in BP in these patients or in the case of a major increase in serum creatinine of more than 30% [75].

### 4.9. Eclampsia and Severe Pre-Eclampsia

Pre-eclampsia can complicate 5 to 7% of pregnancies [76], with a higher rate in women with established hypertension, and an even higher rate in diabetic, first pregnant, multiple fetuses, or hydatidiform mole pregnancies. In pregnant women with pre-existing hypertension, acute worsening of BP control can be due to poor medical treatment or from underlying pre-eclampsia [77]. It is also important to note that pre-eclampsia may develop for the first time intrapartum or postpartum with higher rates of liver impairment and cesarean section delivery rate, while superimposed pre-eclampsia has higher risks of poorer fetal outcome [78].

Management requires a rapid initial lowering of systolic BP in the range of 140 to 150 mmHg and diastolic BP in the range of 90 to 100 mmHg [79]. Medical therapy relies on intravenous labetalol, intravenous nicardipine, or immediate release nifedipine associated with magnesium sulphate for prevention of seizures and convulsions. Monitoring of fetal heart rate is mandatory to avoid bradycardia due to the use of β-blocker therapy.

In a recent meta-analysis, it was demonstrated that oral nifedipine can be considered as a first-line antihypertensive agent for reducing the risk of persistent high BP in pregnancy compared to intravenous hydralazine or labetalol, with no differences in the incidences of adverse effects, maternal or fetal outcomes, or maternal hypotension. For the same purpose, IV hydralazine was more effective than IV ketanserin in controlling BP during pregnancy, with no difference in the risk of maternal hypotension or maternal or fetal outcomes [80].

Another systematic review reported that oral nifepidine has the highest therapeutic success in controlling the BP in pregnancy comparted to IV labetalol or IV hydralazine, while demonstrating no significant difference in the risk of adverse effects between these drugs [81]. 

When the opportunity of switching to oral treatment arises, first choice drugs are methyldopa and long-acting nifedipine. Angiotensin converting enzyme inhibitors, angiotensin-receptor blockers, direct renin-inhibitors, and sodium nitroprusside are contraindicated because of their teratogenic effects, and diuretics should be avoided because they reduce the placental blood flow [10].

### 4.10. Pheocromocytoma/Paraganglioma (PPGL)

An undetected PPGL can have a variety of clinical presentations, including acute heart failure, acute coronary syndrome, aortic dissection, stroke, or eclampsia as first manifestation of the disease. Both plasma and urine tests of free normetanephrine and metanephrine are used for the screening of pheocromocytoma [82]. These plasma and urine tests have a very high negative predictive value, but false positives may occur [82].

α 1 blocking agents, either fentolamine or doxazosin, usually followed by a β blocker, is the first choice for achieving control of BP. The order should be as stated above, to avoid enhanced α1-mediated vasoconstriction. Labetalol is the only β blocker, which also has an α1 blocking effect when administered intravenously, that can be used without prior α1 blockade for the treatment of hypertensive emergencies. Rapidly acting diuretics should be discouraged, as patients suffering from PPGL have relative hypovolemia [83]. 

### 4.11. Acute Perioperative Hypertension and Postoperative Surgical Hypertension

In preoperative patients suffering from chronic hypertension, a thorough evaluation is mandatory to identify possible risk factors for HE. Adrenergic stimulation, postoperative pain, anxiety, and fluctuating intravascular volume can build up towards a perioperative HE. If it is not optimally treated, it can lead to increased bleeding risk and new HMOD [84]. If BP tends to rise above 180/110 mmHg, intensive treatment or delay of surgery is advised to avoid the risk of perioperative or postoperative hypertensive emergency, but every decision needs to be evaluated individually on a case-by-case basis. 

As for the treatment agents, the utility of intravenous clevidipine, nitroglycerin, esmolol, or nicardipine is recognized [85,86]. Clevidipine has been reported as the drug of choice for treating acute postoperative hypertension by a systematic review and meta-analysis, because of its rapid onset and short duration of action, with limited induced variations outside the desired BP and lack of renal or hepatic metabolism. Moreover, it does not show the adverse effects described with nitrates or tachyphylaxis [85]. Esmolol plays an important role in the perioperative setting in cardiac surgery as it can substantially decrease the high burden of supraventricular and ventricular arrhythmias in the aftermath of surgery, with an unclear influence on mortality, acute myocardial infarction, stroke, congestive heart failure, hypotension, and bradycardia. However, in the non-cardiac surgery setting, it can increase the all-cause mortality and stroke rate, but more evidence is needed before a conclusion is to be drawn [87].

### 4.12. Hypertension with Retinopathy

Hypertensive retinopathy is a common finding at fundoscopy and is an important predictor of systemic morbidity and mortality due to HMOD [88]. It has been shown that the increase in incidence of retinopathy is closely related to the degree of duration and severity of uncontrolled hypertension [89].

Flame-shaped hemorrhages and cotton wool spots, which suggest grade III retinopathy, and papillooedema, which suggests grade IV retinopathy, are a sign of microvascular dysfunction and impaired cerebral autoregulation and can be the only signs of an ongoing HE. These advanced forms of hypertensive retinopathy are associated with worse cerebral, renal, and cardiovascular outcome compared with hypertensive patients and no retinopathy [44]. Fundoscopy is therefore an important tool for the evaluation of patients with an acute rise in BP and can lead to faster and better management of HE [90].

## 5. Conclusions

Hypertensive emergencies, while a relatively rare reason for ED presentation, remains a major challenge for the practitioner due to its wide range of clinical patterns, diverse potential A-HMOD, and ability to negatively impact cardiovascular and cerebrovascular outcome. In this educational review, we summarized the current evidence-based knowledge and recommendations for the treatment of HE providing a A-HMOD-centered approach to allow the rapid recognition and proper treatment of these emergencies. The therapeutic strategy, including the magnitude and time course of BP decrease as well as the choice of antihypertensive drugs, should be individualized to the specific type of HE with the scope of limiting organ damage and improving prognosis.

## Data Availability

Not applicable.
